# A Hrd way for MHC-II expression

**DOI:** 10.18632/oncotarget.4799

**Published:** 2015-07-09

**Authors:** Johanna Melo-Cardenas, Sinyi Kong, Deyu Fang

**Affiliations:** Department of Pathology, Northwestern University Feinberg School of Medicine, Chicago, IL, USA

**Keywords:** Immunology and Microbiology Section, Immune response, Immunity

Major histocompatibility complex class II molecules (MHC-II), which present exogenous antigens to CD4^+^ T-lymphocytes, play an essential role in the initiation of the antigen-specific immune response. MHC-II is expressed in antigen presenting cells (APCs) including B cells, monocytes, macrophages and dendritic cells (DCs). MHC-II is constitutively expressed on resting/immature APCs at a relatively lower level, and a variety of inflammatory stimuli dramatically upregulates MHC-II expression at both transcriptional and post-transcriptional levels during infection and autoimmune inflammatory diseases. While extensively studied, the molecular pathways that precisely control MHC-II expression by APCs remain elusive. A recent study uncovered that Hrd1, an endoplasmic reticulum(ER) resident RING (really interesting new gene) finger-containing E3 ubiquitin ligase, is required specifically for the expression of MHC-II by dendritic cells. Genetic deletion of Hrd1 in murine DCs diminished MHC-II expression without affecting their *MHC class I (MHC-I)* expression levels. As a consequence, Hrd1-null DCs failed to prime CD4, but not CD8 T cells both *in vitro* and in mice [1].

Hrd1 promotes MCH-II expression at the transcriptional level because MHC-II mRNA expression is completely abolished in Hrd1-null DCs even upon TLR stimulation. The MHC class II transcription activator (CIITA) has been identified as a critical transcription factor specifically for TLR-induced *MHC-II* transcription in DCs [2]. Interestingly, we discovered that Hrd1 also promotes CIITA expression at the transcription level as *CIITA* mRNA expression was diminished in DCs isolated from *Hrd1^−/−^* mice, indicating that Hrd1 may regulate MHC-II expression by promoting *CIITA* gene transcription possibly by targeting the upstream regulatory factors. Indeed, Hrd1 interacts with BLIMP1, a transcriptional suppressor known to inhibit the expression of both *CIITA* and *MHC-II* [3] to catalyze *BLIMP1* ubiquitination and protein degradation. Loss of *Hrd1* function in DCs resulted in *BLIMP1* protein accumulation and impaired gene transcription of both *CIITA* and *MHC-II,* identifying *Hrd1*-mediated ubiquitination of *BLIMP1* as a positive regulatory pathway in *MCH-II* expression and antigen presentation. It has been shown that *Hrd1* targets the misfolded *MHC-I* molecules for ubiquitination-mediated degradation. However, genetic deletion of *Hrd1* in DCs did not affect *MHC-I* expression, suggesting that *Hrd1* is dispensable in regulating *MHC-I* expression at the physiological condition in the absence of ER stress response. In addition to *Hrd1,* the membrane-associated RING-CH (MARCH) family of E3 ubiquitin ligase I (MARCH I), has been shown to directly bind to and catalyze ubiquitin-conjugation of MCH-II molecules to suppress MHC-II expression by DCs [4]. Therefore, MHC-II expression appears to be regulated by ubiquitin pathways at multiple levels, and the two RING finger-containing E3 ubiquitin ligases Hrd1 and MARCH1 regulate MHC-II expression in an antagonistic fashion.

Hrd1 was initially discovered as an E3 ubiquitin ligase that is responsible for degrading the misfolded proteins accumulated in the ER lumen and to suppress ER stress-induced cell apoptosis. However, loss of Hrd1 function did not yield any ER stress responses in DCs, nor increased DC apoptosis, excluding the possibility that Hrd1 regulates MHC-II expression through ER stress pathway. It was a surprise for us that the ER resident ubiquitin ligase Hrd1 interacts with a nuclear transcriptional suppressor BLIMP1 in mouse DCs. In addition to Blimp1, several transcription factors, including p53, Nrf1, Nrf2 and PGC-1β have been identified as Hrd1 substrates under different physiological or pathological settings [5, 6]. To uncover the molecular puzzles underlying how Hrd1 targets the nuclear transcription factors/suppressors, several critical questions remain to be addressed: First, what are the subcellular localizations of Hrd1 that enable the recognition of its nuclear substrates? Second, is it possible that the ER resident ubiquitin ligase Hrd1 translocates into the nucleus of cells? and third: how is Hrd1 recognition of its nuclear targets regulated by extracellular signaling?

It is still unknown how Hrd1-mediated BLIMP1 ubiquitination is regulated in DCs. Notably, stimulation of DCs with LPS induces Hrd1 gene transcription and enhances Hrd1-BLIMP1 interaction in mouse primary DCs [1]. In addition, proinflammatory cytokines including IL-1β, IL-6, TNF-α and IL-17 have been shown to induce Hrd1 gene expression during inflammatory disease [7]. Therefore, our study identifies a novel pathway that wires TLR/inflammatory cytokine-mediated innate signaling with CD4 T cell-mediated adaptive immunity (Figure 1B). These discoveries imply that Hrd1 is a possible therapeutic target for autoimmune disease treatment. Indeed, a recently identified Hrd1 specific inhibitor that attenuates its ubiquitin ligase activity protected mice from experimental joint inflammatory disease. Moreover, it has been shown that tumor-resident DCs often have dramatically reduced MHC-II expression, implying that tumor cells can evade effector CD4 T cells through modification of DC competence by suppressing MHC-II expression. It will be interesting to study whether Hrd1 is involved in MHC-II expression in tumor resident DCs.

**Figure 1:**
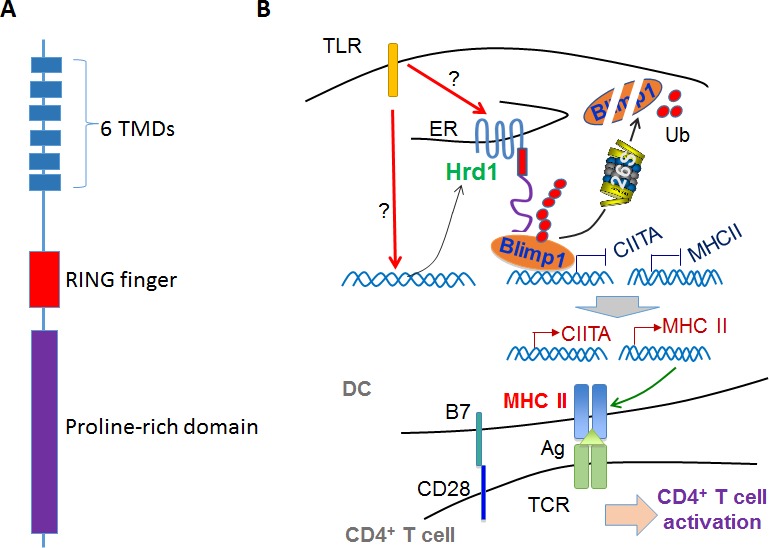
Hrd1 wires innate and CD4 T cell-mediated adoptive immunity **A.** Structure of Hrd1. TMD: transmembrane domain. **B.** A proposed model of Hrd1 in regulation of MHC-II expression. TLR signaling induces Hrd1 gene expression. Hrd1 interacts with and ubiquitinates BLIMP1, a transcription suppresser that inhibits CIITA transcription and consequently to turn on MHC-II expression. MHC-II present foreign antigens to CD4 T cells to initiate the adoptive T cell immune response.

## References

[1] Yang H (2014). J Exp Med.

[2] Steimle V (1993). Cell.

[3] Piskurich JF (2000). Nat Immunol.

[4] De Gassart A (2008). Proc Natl Acad Sci U S A.

[5] Wu T (2014). Genes Dev.

[6] Fujita H (2015). EMBO J.

[7] Gao B (2006). Arthritis Res Ther.

